# Enhanced Performance of Austenitic Oxide Dispersion-Strengthened 316L Steel: A Study on Y_2_O_3_ Reinforcement and Corrosion Behaviour

**DOI:** 10.3390/ma18030641

**Published:** 2025-01-31

**Authors:** Jan Pokorný, Jiří Kubásek, Črtomir Donik, David Nečas, Vojtěch Hybášek, Jaroslav Fojt, Anna Dobkowska, Irena Paulin, Jaroslav Čapek, Matjaž Godec

**Affiliations:** 1 Department of Metals and Corrosion Engineering, Faculty of Chemical Technology, University of Chemistry and Technology in Prague, 160 00 Prague, Czech Republic; necasd@vscht.cz (D.N.); hybasekv@vscht.cz (V.H.); fojtj@vscht.cz (J.F.); 2 Department of Physics and Chemistry of Materials, Institute of Metals and Technology, 1000 Ljubljana, Slovenia; crtomir.donik@imt.si (Č.D.); irena.paulin@imt.si (I.P.); matjaz.godec@imt.si (M.G.); 3 Faculty of Materials Science and Engineering, Warsaw University of Technology, 00-661 Warsaw, Poland; anna.dobkowska@pw.edu.pl; 4 Department of Functional Materials, Institute of Physics of the Czech Academy of Sciences, 182 00 Prague, Czech Republic; capekj@fzu.cz

**Keywords:** mechanical milling, SPS, 316L, austenitic stainless steel, yttria, ODS steel, corrosion, EPR-SL

## Abstract

This study explores the mechanical and corrosion properties of yttria-reinforced 316L stainless steel. Powder precursor materials were prepared using mechanical alloying. Varying yttria (Y_2_O_3_) contents (1, 3, and 5 wt%) were used to assess its impact on the steel’s properties. X-ray diffraction and scanning electron microscopy confirmed the successful dispersion of Y_2_O_3_ within the matrix, with the formation of chromium carbides during spark plasma sintering (SPS). The mechanical properties, including hardness and compressive yield strength, improved with increasing Y_2_O_3_ contents, with the highest strength observed in the 316L-5Y_2_O_3_ sample. However, corrosion resistance decreased with higher yttria concentrations. The 3 wt% Y_2_O_3_ sample exhibited the highest corrosion rate due to localized corrosion in areas enriched with oxide particles and chromium carbides. Electrochemical testing revealed that carbide formation and Cr-depleted regions from SPS processing contributed to the corrosion behaviour. These findings suggest that while yttria reinforcement enhances mechanical strength, optimizing the Y_2_O_3_ content and processing methods is crucial to balance both mechanical and corrosion performance in ODS 316L stainless steel.

## 1. Introduction

Austenitic stainless steels have been extensively investigated for their excellent ductility, toughness, workability, and ability to maintain mechanical integrity at high temperatures. Research from Gelles [1] demonstrated that these steels are widely used across various industries due to these properties, while Kim [2] found that their superior corrosion resistance makes them more favourable than ferritic steels [1,2]. Mathew et al. [3] reported that their performance under high-temperature and high-pressure conditions renders them suitable for demanding applications like nuclear reactors, where materials must endure severe operational environments [3]. However, Xu and Zhou [4] noted that despite these advantages, austenitic stainless steels exhibit limitations, such as relatively low strength and susceptibility to void swelling, particularly when compared to ferritic and ferritic–martensitic steels [4].

To address these challenges, several researchers have focused on the development of oxide dispersion-strengthened (ODS) steels. Murty and Charit [5] emphasized that incorporating fine yttria (Y_2_O_3_) particles into ODS steels significantly enhances mechanical properties by providing pinning points for dislocation motion and grain boundary migration [5]. Akasaka et al. [6] further showed that this mechanism improves yield strength, hardness, creep resistance, and radiation tolerance [6]. Zhao et al. [7] highlighted that the high density of oxide particles acts as sinks for radiation-induced defects, improving swelling resistance. As a result, ODS steels have emerged as promising candidates for structural applications in both fission and fusion reactors, as well as other extreme environments [7].

However, the processing of austenitic ODS steels has presented challenges, particularly due to the stickiness of powders during mechanical alloying, which can reduce milling efficiency and yield. Balázsi et al. [8] discussed this issue, suggesting that it is a significant hurdle in producing high-performance materials [8]. Mechanical alloying (MA) is widely adopted for fabricating ODS steels, and subsequent solid-state consolidation methods, such as hot extrusion (HE) and hot isostatic pressing (HIP), are commonly used [9,10,11,12]. Recent work by Deng et al. [13] highlighted advancements in spark plasma sintering (SPS), which offers advantages in achieving high densification rates at lower temperatures and shorter processing times, thus preventing excessive grain growth and preserving ultrafine oxide dispersion [13].

Despite the growing interest in SPS, research by Yan et al. [14] indicated that the consolidation of ODS powders via SPS is still underexplored. In particular, the effects of varying sintering temperatures and oxide concentrations on the material’s microstructure and mechanical properties remain largely unknown [14]. Furthermore, laser melting technologies like laser powder bed fusion (LPBF) have extensively been studied in recent years in connection to ODS production [15,16,17,18,19].

The aim of this study, as outlined by Wu et al. [20], is to systematically investigate the effects of the Y_2_O_3_ content (1, 3, and 5 wt%) on the microstructural evolution, mechanical properties, and corrosion resistance of 316L ODS steels fabricated via mechanical alloying and SPS [20]. By exploring the interplay between processing parameters, oxide concentration, and performance, the study seeks to optimize the balance between strength and corrosion resistance in these advanced materials, contributing to the development of austenitic ODS steels as high-performance structural materials for extreme environments.

## 2. Materials and Methods

The oxide dispersion-strengthened (ODS) steels were prepared via mechanical alloying (MA) of pre-alloyed 316L austenitic steel and yttria powders at 800 rotation per minute for 1 h in high-energy ball mills. Retsch E-max under argon atmosphere with 0.04 g of stearic acid served as the process control agent (PCA). The milling vessels and balls used were made of AISI 420C steel. The properties of initial powders are shown in Table 1. The ball-to-powder mass ratio was 15:1. The composition of mixed powders was designed as 316L-x Y_2_O_3_ (x = 1; 3; 5 wt%). Y_2_O_3_ was selected due to its proven efficiency in enhancing the mechanical properties of ODS steels. Its high thermal stability and low solubility in the steel matrix ensure the retention of fine, uniformly dispersed oxide particles during high-temperature processing. Among other oxides, such as Al_2_O_3_ or TiO_2_, Y_2_O_3_ has demonstrated superior performance in maintaining dispersion stability and strengthening at elevated temperatures, as documented in prior research. A range of 1–5 wt.% was chosen because it comprises the most effective concentration to optimize dispersion and mechanical properties without leading to excessive microstructural heterogeneity.

The particle size distribution of the powders after mechanical alloying was analyzed using a Malvern Panalytical Mastersizer 3000 laser diffraction system. The data for the individual fractions (D_90_, D_10_ and D_50_) were obtained with relative standard deviations ranging from 1 to 1.5%. The width of the size distribution of particles (span) was calculated based on Equation (1).

Span = (D_90_ − D_10_)/D_50_, (1)

where

D_90_ = particle size below which 90% of the powder volume is present,
D_10_ = particle size below which 10% of the powder volume is present, and
D_50_ = median particle size (mean of distribution).

The mechanically alloyed powders were consolidated by the spark plasma sintering (SPS) method (FCT Systeme HP-D 10) at 1100 °C, 80 MPa for 10 min under vacuum in graphite tools protected by a boron nitride layer.

### 2.1. Microstructure

The prepared samples were first ground on SiC papers P120–P400, then polished on diamond suspension dia-complete 9 μm with polishing cloth Beta, followed by polishing on diamond suspension dia-complete 3 μm with polishing cloth Gamma. Final polishing was executed on the Eposile Non-dry suspension with polishing cloth Zeta. In all cases, metallographical material from QATM was used. The microstructure was characterized by a scanning electron microscope (SEM-Mira II LMU, Tescan, Brno, Czech Republic with an EDS analyzer (EDS-Aztec, Oxford Instruments, Tubney Wood, UK). Electron-backscatter diffraction (EBSD) maps of all the microstructures were collected on a ZEIS Geminy 2 microscope. The phase composition was measured by X-ray diffraction (X’Pert^3^ Powder instrument in Bragg–Brentan geometry using a Co anode (λ = 1.78901, U = 40 kV, I = 35 mA).

### 2.2. Mechanical Properties

The mechanical properties of prepared alloys were characterized by Vickers hardness and compression measurements. The HV1 was measured on a Future-Tech FM-100 at a load of 1 kgf. At least 10 measurements were performed for each sample. Compression tests were performed on cube-shaped specimens with a side length of 2.5 mm at a strain rate equal to 0.001 s^−1^ (Universal testing machine-Instron 5882, Norwood, MA, USA) at 25 °C and 600 °C. At least two measurements were performed for each material.

### 2.3. Corrosion Tests

All electrochemical measurements were performed in a conventional three-electrode setup using a Gamry Instruments electrochemical workstation. The volume of electrolyte per area was 250 mL·cm^−2^.

Austenitic AISI 316L steel produced through conventional methods was used as a reference material. To evaluate the influence of the processing method on corrosion behaviour, a 316L steel sample was also fabricated using powder metallurgy (MA + SPS) under the same conditions employed for the preparation of composites.

Samples were first ground on SiC paper P2500 to achieve reproducible surfaces. The anodic polarization curves were recorded in substituted ocean water at 25 °C, with 1 h stabilization of open circuit potential (Eoc). The potential was measured with respect to the value of ACLE. The scan rate was 1667 mV/s.

The single-loop electrochemical potentiokinetic reactivation test (SL-EPR) was performed in a solution of 0.5 M H_2_SO_4_ + 0.01 M KSCN at 25 °C. The specimens were polished using a 2 μm diamond paste. The surface of the samples was first potentiostatically passivated by applying the potential of +200 mV/ACLE and holding for 2 min. Subsequently, the sample was reverse polarized from +200 mV/ACLE to a potential of 50 mV below the E_OC_ with a scan rate of 3 mV/s. For each sample, the microstructure was characterized after electrochemical measurements. Next, the charge (Q) was evaluated by integrating it as the area under the curve. Using this value and the appropriate Equation (2) based on the standard, the normalized charge (Q_Pa_) was determined.

Q_Pa_ = Q/X, (2)

where

Q = charge measured on current integration measuring instrument (C),
X = A_s_ [5.1 × 10^−3^·*e*^0.35^·G],
A_s_ = specimen area (cm^2^), and
G = grain size number ().

## 3. Results

### 3.1. Powder Precursors

**Figure 1:**
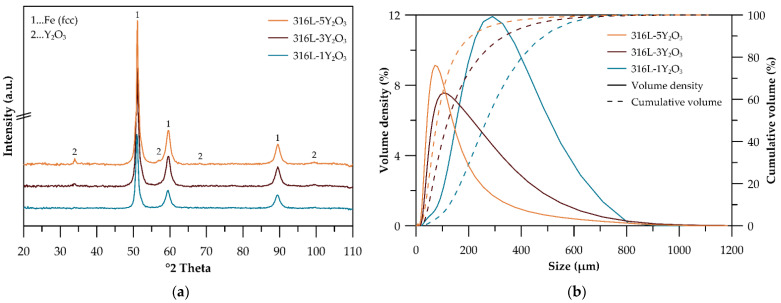
(**a**) XRD patterns of MA powders; (**b**) particle size distribution of MA powders.

**Figure 2:**
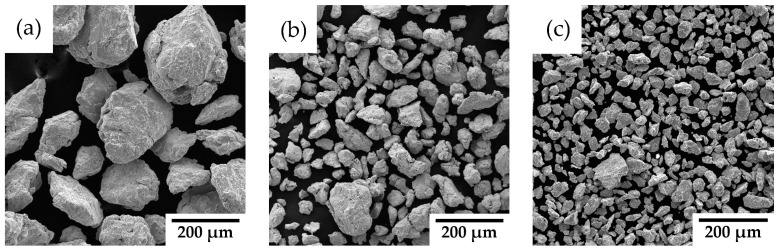
SEM micrographs of mechanically alloyed powders: (**a**) 316L-1Y_2_O_3_; (**b**) 316L-3 Y_2_O_3_; (**c**) 316L-5 Y_2_O_3_.

Mechanical alloying with a process control agent was selected as the optimal method for preparing the powder alloy to prevent the undesirable sticking of the powder to the milling balls and vessel walls during high-energy milling. Figure 1a presents the XRD spectra of materials processed at 800 RPM for 1 h with varying Y_2_O_3_ contents (1, 3, and 5 wt%). The dominant peaks correspond to the FCC structure of 316L stainless steel. Peaks attributable to Y_2_O_3_ are also evident, signifying the presence of oxide particles. These Y_2_O_3_-specific peaks become increasingly pronounced with higher Y_2_O_3_ contents, particularly in the 3 and 5 wt% samples, indicating a greater fraction of the oxide phase. The analysis thus confirms the coexistence of the FCC austenitic matrix and Y_2_O_3_ oxide phases. Figure 1b illustrates the particle size distribution of the mechanically alloyed powders. The mechanically alloyed powders of all alloys exhibit polymodal distribution curves, indicating the presence of both fine and coarse particles. A clear trend of powder refinement with increasing Y_2_O_3_ concentration is observed, further confirmed by the scanning electron microscope images in Figure 2. Table 2 summarizes the characteristic distribution metrics for each alloy, including D_50_, D_90_, D_10_, and the span, according to Equation (1).

### 3.2. Consolidated Materials

**Figure 3:**
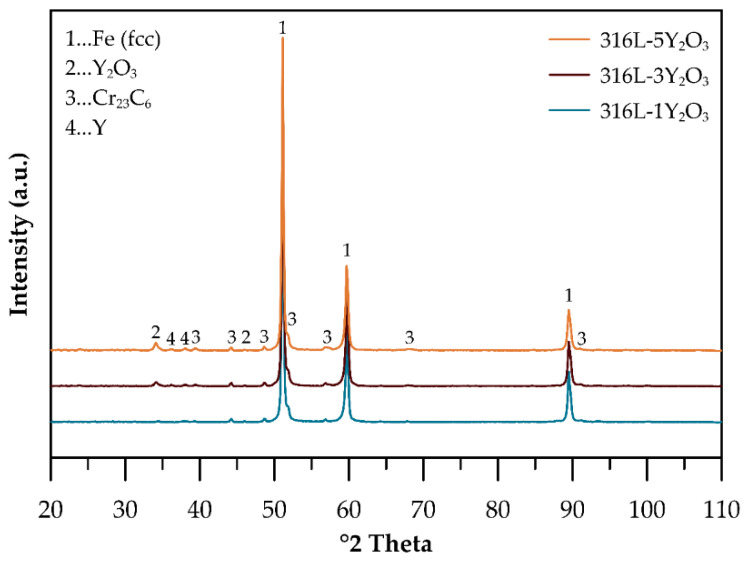
XRD patterns of consolidated samples.

The phase composition of consolidated ODS steel samples reinforced with 1, 3, and 5 wt% Y_2_O_3_ was analyzed using XRD, as shown in Figure 3. The XRD patterns of all samples consolidated by SPS at 1100 °C confirm the retention of the FCC austenitic structure as the dominant phase. Peaks corresponding to Y_2_O_3_ are clearly visible. These peaks increase in intensity with higher Y_2_O_3_ contents, reflecting the growing oxide fraction. Additionally, the appearance of new peaks suggests the significant formation of chromium carbides during the SPS process. Surprisingly, part of the Y in the microstructure is according to the XRD presented as a pure element. Although the peaks of Y are not clearly evident in the XRD spectra of powders, this can be related to the generally low content of metallic Y phase. We believe that yttrium may be partially formed during the milling process by the breakage and dissolution of Y_2_O_3_ into the solid solution.

**Figure 4:**
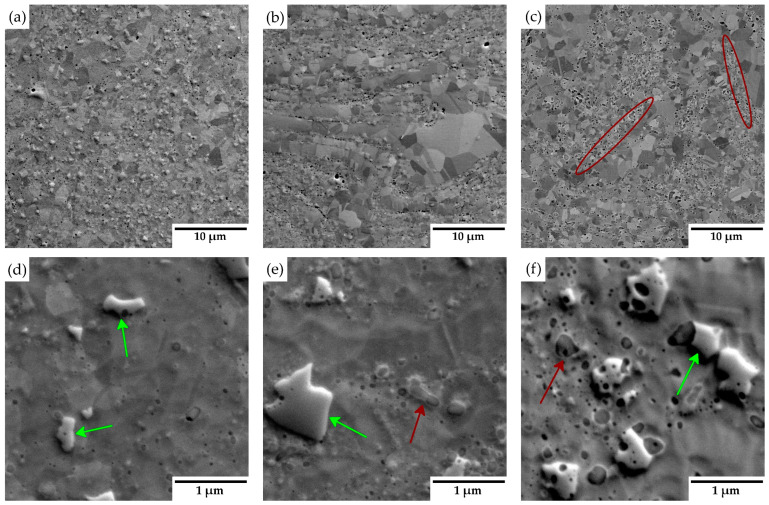
(**a**–**c**) General microstructure of consolidated materials (red ellipses-Y_2_O_3_ bands); (**d**–**f**) close-up images of consolidated materials (red arrows—oxide particles; green arrows—Cr_23_C_6_), with varying yttria contents: (**a**,**d**) 1 wt %; (**b**,**e**) 3 wt %; (**c**,**f**) 5 wt %.

The microstructure of the consolidated samples is presented in Figure 4. No obvious porosity was detected in the materials, indicating a formation of materials of almost theoretical density. Scanning electron microscope images reveal a relatively uniform distribution of Y_2_O_3_ particles throughout the matrix. Additionally, regions with locally coarse grains and varying grain sizes are visible. Close-up images in Figure 4d–f highlight the presence of Cr_23_C_6_ (green arrows) and oxide particles (red arrows), predominantly located at grain boundaries. In samples with higher Y_2_O_3_ concentrations (3 and 5 wt.%), bands with increased Y_2_O_3_ contents are observed, indicating localized enrichment in these regions (highlighted in red ellipses).

**Figure 5:**
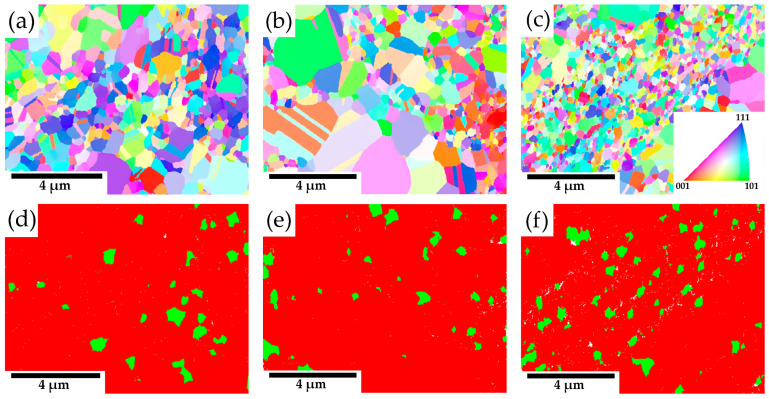
(**a**–**c**) EBSD IPF maps of consolidated materials; (**d**–**f**) phase distribution maps of consolidated materials—carbides are in green, austenite in red, with varying Y_2_O_3_ contents: (**a**,**d**) 1 wt %; (**b**,**e**) 3 wt %; (**c**,**f**) 5 wt %. IPF maps corresponds to the Z axis which is parallel to the vertical axis of the SPSed sample. Both austenite and carbide phase have similar fcc structure; therefore, they are not distinguished in the IPF map.

Figure 5a–c presents EBSD IPF maps of compacted samples with varying concentrations of Y_2_O_3_. The materials exhibit a randomly oriented grain structure. Furthermore, all samples display a bimodal grain size distribution, comprising a mix of ultrafine (up to 0.6 µm) and coarse grains (up to 2.8 µm). Grain twinning, a characteristic feature of austenitic steels, is also observed in the microstructures, especially in the larger grains. Phase distribution maps in Figure 5d–f highlight the presence of chromium carbides (highlighted in green) within the microstructure. The amount of carbides estimated by area fraction from EBSD is almost similar for all materials and corresponds to the range of 5–6.5%.

### 3.3. Mechanical Properties

The mechanical properties were assessed for austenitic steel samples reinforced with 1, 3, and 5 wt% Y_2_O_3_. A conventionally produced 316L steel specimen was used as the reference standard for Vickers hardness measurements. For compression testing, a 316L steel sample fabricated via powder metallurgy as other composites served as the reference.

**Figure 6:**
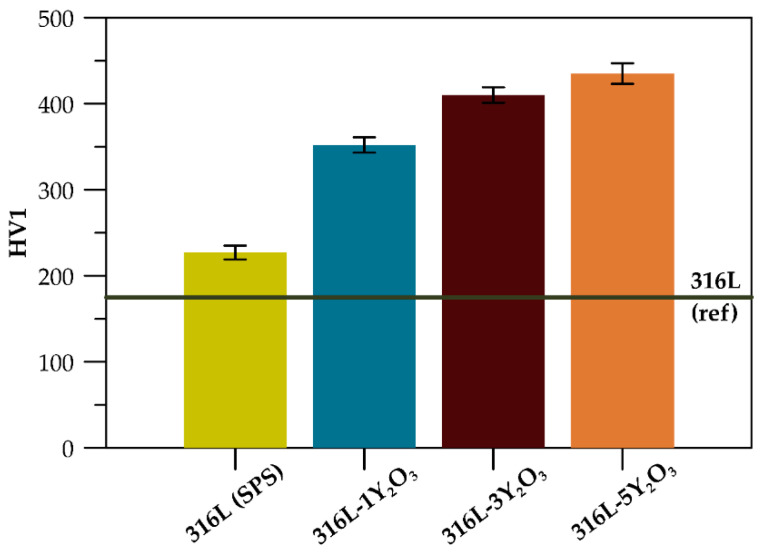
Hardness chart of consolidated materials.

A comparison of the mechanical properties reveals variations in hardness (HV1) and compressive yield strength across materials with different reinforcement concentrations. Hardness increases progressively with higher Y_2_O_3_ contents (Figure 6), with the 316L-5Y_2_O_3_ specimen achieving the highest value of 435 HV1. Relative to the reference 316L steel, hardness showed an increase of 30% for the SPS-processed 316L sample, 101% for 316L-1Y_2_O_3_, 134% for 316L-3Y_2_O_3_, and 149% for 316L-5Y_2_O_3_.

**Figure 7:**
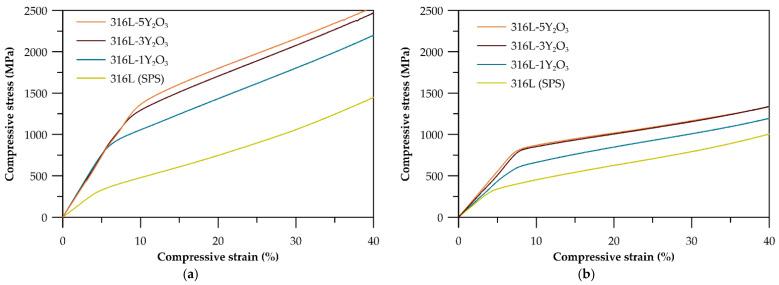
(**a**) Stress–strain curves of austenitic ODS steel at 25 °C for varying Y_2_O_3_ contents; (**b**) stress–strain curves of austenitic ODS steel at 600 °C for varying Y_2_O_3_ contents.

The compressive tests conducted at room temperature (Figure 7a) reveal a significant difference in mechanical properties between materials with 1 wt% and 3 wt% Y_2_O_3_ reinforcement. All materials exhibit substantial plasticity, with deformations exceeding 40%, where the test was manually stopped. Samples with 5 wt% reinforcement show properties comparable to those with 3 wt%. The highest compressive yield strength of 1035 MPa was recorded for the 316L-5Y_2_O_3_ sample, while the lowest value (775 MPa) was observed for the 316L-1Y_2_O_3_ sample. Compared to the SPS-processed reference material 316L, the yield strength increased by 147% and 230% for the 1 wt% and 5 wt% reinforced samples, respectively. The yield strength values are further summarized in Table 3, providing a clear comparative overview of the mechanical performance across all tested materials. At an elevated temperature of 600 °C (Figure 7b), the yield strength of all materials decreased compared to the results observed at room temperature. The stress–strain curves for the specimens with 3 and 5 wt% Y_2_O_3_ reinforcement exhibited nearly identical patterns, with similar yield strength values and overall stress–strain behaviour. The material with 1 wt% Y_2_O_3_ demonstrated significantly higher yield strength than the reference 316L (SPS).

### 3.4. Corrosion Tests

#### 3.4.1. Potentiodynamic Curves

**Figure 8:**
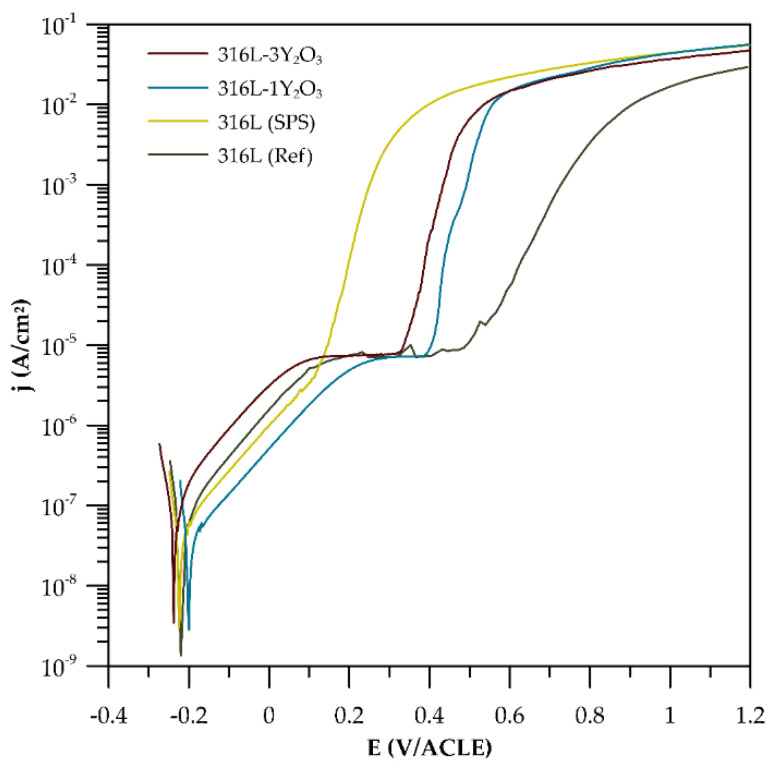
Potentiodynamic curves.

The potentiodynamic polarization curves were measured for 316L stainless steel produced by SPS and cast and for ODS steel reinforced with 1 and 3 wt% Y_2_O_3_. As shown in Figure 8, all curves exhibit a similar trend, indicating that materials underwent passivation. The steep slope of the cathodic tafel region further indicates the oxidation process and formation of a passive layer. It is worth underlining that the highest resistivity to localized corrosion was observed in the reference sample, which is proven by the ∆E (the difference between E_corr_ and E_b_) shown in Table 4. On the contrary, the lowest resistivity to localized corrosion was observed for 316L (SPS). ODS steels are characterized by higher E_b_, although ∆E is reduced with a higher amount of Y_2_O_3_ in materials.

#### 3.4.2. EPR-SL

**Figure 9:**
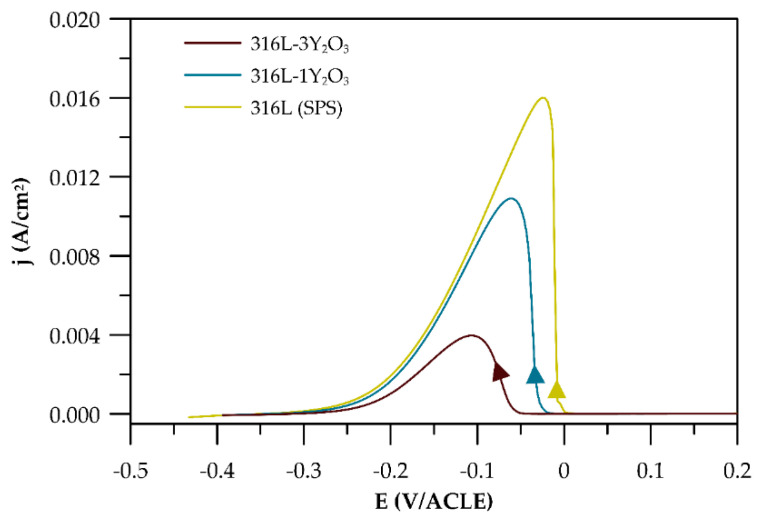
EPR-SL curves of consolidated materials.

**Figure 10:**
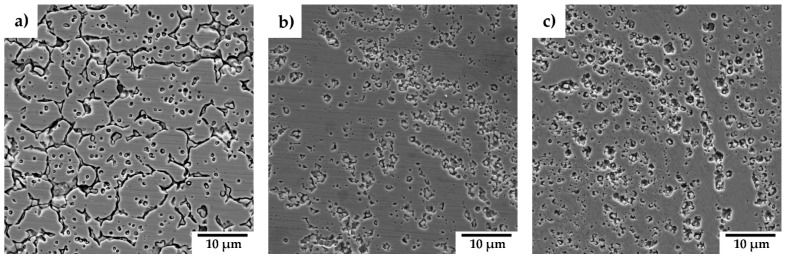
SEM surface micrographs of the materials after exposure: (**a**) 316L (SPS); (**b**) 316L-1Y_2_O_3_; (**c**) 316L-3Y_2_O_3_.

The electrochemical potentiokinetic reactivation curves obtained using the single-loop method (Figure 9) show a clear increase in the reactivation peak current density as the Y_2_O_3_ content in the materials decreases. From these curves, the reactivation charge Q (C) was calculated for each material and normalized to the surface charge density Q_Pa_ (C/cm^2^), as summarized in Table 5. The degree of sensitization was observed to increase with decreasing Q_Pa_. Surface micrographs of the materials after exposure (Figure 10) reveal preferential corrosion along grain boundaries and around chromium carbides in the case of SPS-processed 316L steel, corresponding to Cr-depleted regions in the matrix. In materials containing Y_2_O_3_, localized corrosion predominantly occurs in areas with elevated concentrations of Y_2_O_3_ particles, as shown in Figure 10. The micrographs highlight these regions where corrosion is most prominent, particularly at the interfaces where Y_2_O_3_-rich areas overlap with regions enriched in carbides. This correlation is supported by the electrochemical potentiokinetic reactivation (EPR) results, which indicate a higher degree of sensitization in these areas. The clustering of Y_2_O_3_ particles, combined with chromium depletion near carbides, creates localized microstructural heterogeneities that act as preferential sites for corrosion initiation.

## 4. Discussion

### 4.1. Powder Precursors

The XRD spectra in Figure 1a verify the presence of the FCC structure in all powders, demonstrating the retention of the austenitic phase following mechanical alloying. Notably, the primary FCC peaks remain largely unchanged with the addition of Y_2_O_3_, suggesting that the oxide particles are well dispersed and have minimal impact on the bulk matrix structure. The polymodal particle distribution shown in Figure 1b offers a distinct advantage for the preparation of consolidated samples via spark plasma sintering, as fine particles enhance bulk density and reduce porosity by filling the voids between coarse particles, a phenomenon similarly observed for SiC materials in the work of S. Lemonnier et al. [21]. The particle distribution width, expressed as a span in Table 2, indicates that a higher span value corresponds to a broader distribution and improved packing density. Accordingly, samples with 3 and 5 wt% Y_2_O_3_, which have span values of 2.4 and 2.2, respectively, demonstrate better bulk density compared to the 1 wt% Y_2_O_3_ sample, which exhibits a narrower particle distribution. Particle size analysis (Figure 1b) demonstrates a distinct trend of powder refinement with increasing Y_2_O_3_ concentrations, a finding further supported by the scanning electron microscope (SEM) images in Figure 2. The powders exhibit irregular and angular particle shapes, resulting from the cold welding and subsequent fracturing of particles during mechanical alloying. As in our case, Wang M. et al. [22] also observed particle downsizing caused by strain accumulation and work hardening during mechanical milling, which ultimately led to particle embrittlement and fracture. With the Y_2_O_3_ content increasing from 1 wt.% to 5 wt.%, the powders display a polymodal size distribution alongside a noticeable reduction in average particle size (Table 2). This refinement can be attributed to the mechanical milling process, where the hard Y_2_O_3_ oxide particles facilitate the fragmentation of larger particles formed through cold welding, thereby enhancing the homogeneity of yttrium distribution in the powder.

### 4.2. Consolidated Materials

The X-ray diffraction analysis of the consolidated materials (Figure 3) identifies an additional phase alongside the austenitic matrix and yttrium oxides: chromium carbides [23]. The formation of these carbides likely occurs unintentionally during the consolidation process, resulting from carbon introduction in the powder during mechanical alloying due to the use of a process control agent (PCA) and the subsequent diffusion of carbon from graphite dies and punches. Microstructure images in Figure 4 reveal areas with locally coarsened grains, likely resulting from localized thermal effects occurring between powder particles during the consolidation process. These thermal gradients can promote grain growth in specific regions, leading to the observed coarsening [24]. The phenomenon of locally coarsened grains is also evident in the IPF maps from the EBSD analyses presented in Figure 5a–c and was also observed by Ninawe et al. [25]. Additionally, as the Y_2_O_3_ concentration increases, bands with higher concentrations of oxide particles become evident. This distribution is likely caused by the clustering tendency of Y_2_O_3_ particles during mechanical alloying. These areas are more susceptible to material removal, which can lead to the formation of additional pores. In addition, the polishing process may release loosely bound particles from the surface, particularly in areas where the matrix/oxide interface is weak or where oxide agglomeration is prevalent. These effects can obscure the visual appearance of pores in the prepared samples. In such areas, the grain size is significantly reduced because the particles pin the grain boundaries and prevent their migration. Bimodal grain size distributions have also been observed in other studies on SPS-consolidated alloys [10,20,26].

### 4.3. Mechanical Properties

As mentioned in Section 3.3., the hardness of the materials increases with rising Y_2_O_3_ content, primarily due to the hardening effect of the oxide particles. While the precipitated carbides also influence hardness, their consistent abundance and distribution across all materials ensure that their impact does not overshadow the effect of the Y_2_O_3_ particles. Koul et al. also reported an increase in hardness with higher Y_2_O_3_ oxide content [27]. Additionally, the graph in Figure 6 highlights an improvement in hardness for the SPS-processed sample compared to the commercially produced reference sample. This increase is attributed to the presence of carbides in the microstructure and grain size refinement achieved through the SPS process.

The compressive behaviour of ODS 316L steel at both room temperature and elevated temperatures (600 °C) demonstrates the good material’s plasticity under compression. The yield strength of ODS 316L (SPS) exceeds that of conventional 316L stainless steel both at laboratory and elevated temperatures. This enhanced yield strength is attributed to the grain refinement and bimodal grain size distribution, the strengthening effect of dispersed Y_2_O_3_, but also the effect of carbide particles. Despite the presence of carbides, all tested materials exhibit significant compressive strain capacity, with compressive strains exceeding 40%. The compression curves at room temperature, shown in Figure 7a, reveal that even a modest addition of 1 wt% Y_2_O_3_ results in a notable increase in yield strength, which is also affected by carbides. As the Y_2_O_3_ content rises to 3 and 5 wt%, the yield strength continues to improve [27]. Due to the similar content of carbides in materials, such an increase has to be related to the Y_2_O_3_ content in the microstructure. However, the incremental strengthening effect between the 3 wt% and 5 wt% materials is less pronounced, most probably due to the more significant formation of clusters of Y_2_O_3_ and, therefore, the increased material heterogeneity. At the elevated temperature of 600 °C, the compression curves (Figure 7b) show a change in slope in the elastic region compared to the room temperature curves, which is attributed to a decrease in the elastic modulus (E). However, the precise value of *E* could not be determined due to the absence of a tensometer during the measurements. Yield strengths are lower across all materials at this temperature. Notably, the strengthening effect of 5 wt% Y_2_O_3_ is reduced at elevated temperatures, with the stress–strain curve for this sample closely resembling that of the 3 wt% Y_2_O_3_ material. This phenomenon can be attributed to thermal softening effects, which become more pronounced at elevated temperatures, partially offsetting the strengthening contribution of the additional Y_2_O_3_ in the 5 wt% sample. At higher oxide concentrations, the tendency for particle clustering and agglomeration increases, reducing the effectiveness of the oxide dispersion and limiting further mechanical improvement. Additionally, the elevated temperature may facilitate grain boundary sliding, diminishing the impact of the oxide particles on dislocation pinning and grain boundary strengthening. These factors collectively explain the convergence of mechanical properties between the 3 wt% and 5 wt% Y_2_O_3_ samples under high-temperature conditions.

### 4.4. Corrosion Tests

The findings on the basic corrosion behaviour, as outlined in Section 3.4 and derived from the potentiodynamic curves in Figure 8, reveal that corrosion resistance is significantly influenced by both the processing method and the Y_2_O_3_ content. 316L steel prepared via SPS exhibits the lowest corrosion resistance, with a breakdown potential of just 116 mV—markedly lower than that of the reference cast material. This diminished performance is attributed to the formation of carbides and Cr-depleted grain boundary regions during the SPS process, which serve as preferential sites for localized corrosion. Pardo et al. [28] as well highlighted that the corrosion resistance of austenitic stainless steels in chloride-containing environments primarily arises from the enrichment of the chromium oxide layer at the metal–environment interface. However, during compaction, the formation of chromium carbides leads to chromium depletion in the adjacent grain boundary regions, adversely affecting corrosion resistance. These results underscore the adverse impact of processing-induced microstructural inhomogeneities on corrosion resistance. For the Y_2_O_3_-reinforced samples, corrosion resistance decreases as the Y_2_O_3_ content increases. Although Y_2_O_3_ particles can act as stabilizers of the passive layer [29], localized corrosion predominantly occurs in regions enriched with these oxide particles. This effect is especially pronounced at interfaces where carbides and Cr-depleted zones coexist [30]. The presence of such features indicates that regions with higher Y_2_O_3_ concentrations introduce microstructural heterogeneities that facilitate localized corrosion. Interestingly, the sample with 1 wt% Y_2_O_3_ demonstrates a lower corrosion rate compared to the sample with 3 wt% Y_2_O_3_. Among all the materials tested, the 3 wt% Y_2_O_3_ sample exhibits the highest corrosion rate. This elevated rate is likely due to the increased number of oxide phases that serve as active sites for corrosion initiation. These observations suggest that while Y_2_O₃ provides mechanical benefits, its content must be carefully optimized to balance mechanical and electrochemical performance.

The EPR-SL method results, illustrated in Figure 10, reveal distinct mechanisms of localized corrosion, aligning with the findings from the potentiodynamic curves. For the 316L sample (Figure 10a), localized corrosion primarily occurs along grain boundaries and around carbides, where chromium depletion leads to increased susceptibility to corrosion attack [31,32]. In contrast, the Y_2_O_3_-reinforced samples (Figure 10b,c) exhibit a different corrosion mechanism. Corrosion in these materials predominantly affects the matrix surrounding carbides and regions with a higher oxide content. This mechanism is reflected in the greater degree of desensitization observed for the sample containing 3 wt% Y_2_O_3_, consistent with the electrochemical results in Table 5. The microstructures of these hardened materials feature regions with both very fine grains and coarse grains. Notably, the very fine grains are concentrated in areas rich in yttrium oxides, which act as sites for localized corrosion. Consequently, these regions are particularly susceptible to grain corrosion and pitting, further contributing to the observed degradation patterns. This underscores the role of microstructural features and oxide dispersion in influencing the corrosion behaviour of these materials.

While Y_2_O_3_ reinforcement significantly enhances the mechanical properties of 316L stainless steel, the increased susceptibility to localized corrosion, particularly at higher Y_2_O_3_ contents, raises concerns about its suitability for certain nuclear power engineering applications. The presence of Cr-depleted zones and oxide clustering could compromise the material’s long-term durability in aggressive environments typical of nuclear systems.

However, with optimized processing parameters and improved oxide dispersion techniques, these challenges may be mitigated, potentially making Y_2_O_3_-enriched 316L steel a viable candidate for nuclear applications requiring high strength and radiation resistance. Future research should focus on addressing these limitations to fully realize the material’s potential in such demanding environments.

## 5. Conclusions

This paper demonstrates the study of Y_2_O_3_-strengthened 316L stainless steel with enhanced mechanical properties, which persist despite some compromise in corrosion resistance. The key findings are summarized as follows:

(1) Mechanical alloying effectively produced homogeneous 316L powders reinforced with Y_2_O_3_.
(2) Spark plasma sintering preserved the FCC austenitic structure but introduced chromium carbides and microstructural heterogeneities at higher Y_2_O_3_ concentrations, influencing mechanical and corrosion behaviour.
(3) Increasing Y_2_O_3_ contents enhanced hardness and compressive yield strength while maintaining compressive plasticity, with diminishing returns at contents above 3 wt%.
(4) At elevated temperatures, mechanical performance decreased; however, composites still exhibited significantly higher compressive yield strength compared to the reference 316L steel.
(5) Corrosion resistance was compromised by localized oxide-rich regions and Cr-depleted zones, particularly at higher Y_2_O_3_ contents.
(6) Localized corrosion was more prominent in areas of Y_2_O_3_ clustering and carbide enrichment.

## Figures and Tables

**Table 1:** Properties of initial powders.

Initial Powders	Purity (%)	Size (μm)
Pre-alloyed 316L steel	99.99	30–60
Y_2_O_3_	99.9	1–2

**Table 2:** Particle size distribution parameters for alloys with different compositions.

Alloy	D_90_ (μm)	D_10_ (μm)	D_50_ (μm)	Span
316L-1Y_2_O_3_	486.8	126.9	267.7	1.4
316L-3Y_2_O_3_	336.3	48.0	121.6	2.4
316L-5Y_2_O_3_	209.7	35.6	80.3	2.2

**Table 3:** Compressive yield strength (CYS) of ODS steels in compression at room and elevated temperatures (600 °C) and Vickers hardness.

Material	CYS_0.2_ (MPa)	CYS_0.2_ 600 °C (MPa)	HV1
316L (SPS)	313 ± 9	296 ± 7	227 ± 8
316L-1Y_2_O_3_	775 ± 21	550 ± 32	352 ± 9
316L-3Y_2_O_3_	1022 ± 15	803 ± 9	410 ± 9
316L-5 Y_2_O_3_	1042 ± 7	835 ± 6	435 ± 12

**Table 4:** Electrochemical parameters of austenitic ODS steel samples.

Material	E_corr_ (mV/ACLE)	β_c_ mV/Decade	E_b_ (mV/ACLE)	∆E (mV)
316L (ref)	−220	166.8	472	252
316L (SPS)	−225	177.7	116	109
316L-1Y_2_O_3_	−200	180.5	395	195
316L-3Y_2_O_3_	−235	178.9	328	93

**Table 5:** Results of the SL-EPR test and calculation of Q_Pa_.

Material	Q (C)	X (cm^2^)	Q_Pa_ (C/cm^2^)
316L (SPS)	0.068	0.029	20.7
316L-1Y_2_O_3_	0.025	0.043	09.0
316L-3Y_2_O_3_	0.007	0.043	03.1

## Data Availability

The data presented in this study are openly available in ZENODO with DOI 10.5281/zenodo.14622099.
